# Principles for Evaluating the Efficacy and Safety of Ceramic Dental Implants in Japan

**DOI:** 10.1007/s43441-024-00713-7

**Published:** 2024-11-05

**Authors:** Tomoya Hara, Yuchi Sato, Hiroyuki Tanishiro, Yukimichi Tamaki, Shunsuke Baba, Eiichi Hirose, Bunsaku Yoshida, Kiyoshi Watanabe, Genki Nishikawa, Daiju Okuda, Madoka Murakami, Yuki Niwa, Masuo Kondoh

**Affiliations:** 1 Office of Medical Devices II, Pharmaceuticals and Medical Devices Agency, Tokyo, 100-0013 Japan; 2 Translational Research Institute for Medical Innovation, Osaka Dental University, Osaka, 573-1121 Japan; 3 Department of Dental Materials Science, Division of Oral Functional Science and Rehabilitation, Asahi University School of Dentistry, Gifu, 501-0296 Japan; 4 Department of Oral Implantology, Osaka Dental University, Osaka, 573-1121 Japan; 5 Japan Association of Dental Implant Suppliers, Tokyo, 108-0014 Japan; 6 Japan Dental Materials Manufacturers Association, Tokyo, 111-0056 Japan; 7 Pharmaceutical Safety and Environmental Health Bureau, Ministry of Health, Labour and Welfare, Tokyo, 100-8916 Japan; 8 Graduate School of Pharmaceutical Sciences, Osaka University, Osaka, 567-0871 Japan; 9 Present Address: Healthy Policy Bureau, Ministry of Health, Labour and Welfare, Tokyo, 100-8916 Japan; 10 Present Address: Japan Agency for Medical Research and Development, Tokyo, 100-0004 Japan; 11 Present Address: Office of Software as a Medical Device, Pharmaceuticals and Medical Devices Agency, Tokyo, 100-0013 Japan

**Keywords:** Dental Implant, Ceramics, Safety, Efficacy, Material

## Abstract

Recent progress in materials chemistry has resulted in the development of several ceramic materials that are now being used in dental implants. The advantages of ceramic materials over conventional metallic materials are that they do not induce allergic reactions in individuals with metal allergies, they do not interfere with magnetic resonance imaging, and they provide improved esthetics. In addition, some ceramic materials are tougher than metallic materials and less brittle. However, despite these advantages, few ceramic dental implant materials are currently approved for use in Japan. In FY2022, the Ministry of Health, Labour and Welfare of Japan commissioned a project called the “Project for the Development of a Guideline for the Evaluation of Ceramic Dental Implants,” the goal of which was to consider how best to facilitate swift clinical development and approval of emerging ceramic dental implant materials. At a meeting of experts from professional societies, related industry organizations, and government agencies, the issues related to evaluation of the efficacy and safety of ceramic implant were discussed. Here, we summarize the outcomes of that meeting as a set of principles for the premarketing evaluation of ceramic dental implant materials in Japan.

## Introduction

Dental caries and periodontal disease are non-communicable oral diseases and risk factors for tooth loss. The burden of these oral diseases increases with aging [1]. Dental implants are a therapeutic option to restore functional loss of mastication and improve esthetics in individuals without teeth. The current gold-standard material for dental implants is titanium and its alloys; however, these materials can induce allergic reactions in individuals with metal allergies and can have poor esthetics. To address these issues, ceramic implant materials such as zirconia have been developed [2]. Zirconia is superior to titanium with respect to osteoblastic adhesion and proliferation; moreover, it does not induce allergic reactions in individuals with metal allergy and it has better material fatigue and esthetic properties, although it has lower ductility and a lower elastic modulus than does titanium [2, 3].

Despite the advantages of ceramic implant materials, few such materials are currently approved for use in Japan. In FY2022, the Ministry of Health, Labour and Welfare of Japan commissioned a project called the “Project for the Development of a Guideline for the Evaluation of Ceramic Dental Implants,” the goal of which was to consider how best to facilitate swift clinical development and approval of emerging ceramic dental implant materials. At a meeting of experts from professional societies, related industry organizations, and government agencies, the issues related to the evaluation of efficacy and safety of ceramic implant materials were discussed. In this document, we summarize the outcomes of this meeting as a set of principles for the premarketing evaluation of ceramic dental implant materials in Japan.

## General Matters

In this document, the term “dental implants” is used to refer to medical devices with the generic names of “dental endosseous implant” “dental implant fixtures,” “dental implant systems,” and “dental implant abutments,” as specified in the Japanese Act on Securing Quality, Efficacy and Safety of Products Including Pharmaceuticals and Medical Devices (The Pharmaceutical and Medical Device Act) [4]. Definitions of the other terms used in this document are provided in Table 1. In addition, it should be noted that this document covers only dental implants made from ceramic materials containing zirconia as the main ingredient. For dental implants made of ceramic materials other than zirconia, the applicability of the principles contained herein should be considered with respect to the individual properties of these other ceramic materials.

**Table 1:** Definitions of terms used in this document

Term	Definition
Dental implant	A medical device made of biocompatible materials that is surgically implanted in, or directly connected to, the maxilla or mandible to restore masticatory function. In this document, this term refers to dental endosseous implants, dental implant fixtures, dental implant systems, and dental implant abutments.
Dental endosseous implant	A dental implant that is partially or totally implanted in the jaw bone. In this document, this term includes dental implant fixtures and dental implant abutments.
Dental implant fixture	The part of a dental implant that is surgically implanted into bone. Screw-type and cylinder-type fixtures are available.
Dental implant abutment	An element that is fixed to the dental implant fixture to form an abutment of the superstructure or used temporarily until the gingiva heals. In this document, this term includes the abutment screw used for fixing the dental implant abutment.
Dental implant system	A system consisting of dental implants, instruments for implant surgery, and laboratory instruments used to fabricate the superstructure. In this document, this term refers only to a system composed of a dental implant fixture and a dental implant abutment.
Immediate loading	A procedure involving implantation of a dental endosseous implant material or dental implant fixture and subsequent occlusal loading of a prosthesis within the following 48 h.
Early loading	A procedure involving implantation of a dental endosseous implant material or dental implant fixture and subsequent occlusal loading of a prosthesis onto the dental implant before bone healing (which requires 4 months for the maxilla and 3 months for the mandible). Immediate loading is excluded.
Temporary implant	A dental endosseous implant or dental implant fixture that is not intended for permanent use.
Surface treatment	A process applied to a part of, or the entire surface of, a dental implant. In this document, this term is used in reference to a product that has been manufactured and has undergone some surface processing on the manufactured surface.

The premarketing evaluation of ceramic dental implants (hereafter, “the product”) should address three overarching points. First, the uniqueness, improvements, and equivalence of the product compared with current approved products should be clarified. For example, it should be clarified that the product is equivalent to currently approved titanium dental implants, or that it is superior to currently approved titanium dental implants in terms of immediate loading and early loading, or that is addresses an unmet medical need. If an esthetics claim related to the color tone is made, the applicant should clarify the clinical significance of the color tone and then evaluate the esthetic property of the product. Similarly, if a claim of a low risk of inducing allergic symptoms to metal is made, the applicant should confirm the absence of metal in the material or the degree to which any metal is present.

Secondly, the method of using the product to ensure adequate safety should be established, particularly when the safety concerns differ from those for currently approved titanium dental implants. For example, for dental implant fixtures, the method of forming an insertion socket and inserting the product should be clarified. When dental implant abutments are connected to dental implant fixtures, and when dental implant superstructure materials are connected to dental implant abutments, the tightening method and the recommended torque should be established. The points to consider in cases in which removal of the dental implant fixture is required should also be established.

Thirdly, the concomitant use of any medical devices should be clarified. For example, superstructures should be identified by information such as the brand name and certification number. When a dedicated instrument is used for implanting a dental fixture or fastening an abutment, the instrument should be identified by information such as the brand name, generic name, and certification/notification number. If the instrument to be used is not identified as a dedicated instrument, it is acceptable to specify the conditions required for the medical device to be used and to provide information such as any critical specifications and the generic name of the instrument.

### Evaluation of Quality, Safety, and Performance

Within the categories of quality, safety, and performance, there are a number of individual items that should be evaluated. These items are summarized below and in Table 2. The evaluations should be performed by using raw materials or a test sample prepared under the same conditions as the final product [5, 6].

**Table 2:** Items for premarketing evaluation of ceramic dental implant materials in Japan

Category	Item
Safety	• Biological safety [6, 14, 15] • Magnetic resonance safety [16]
Quality	• Raw materials • Chemical composition • Fine structure (mean crystal grain size) • Surface treatment • Stability [20, 21] • Hot-water stability • Assurance of sterility • Residual ethylene oxide testing [11] • Risk assessment [12, 13] • Packaging
Performance	• Surface roughness [22] • Bending strength [7] • Fatigue strength [17, 18] • Fracture toughness [19, 23] • X-ray detectability [24, 25] • Solubility and degradability [19] • Evaluation required according to the characteristics of the product
Other	• Simulated-use test [15]

#### Quality

##### Raw Materials

Such materials should fulfill the standards of either ISO 13,356 or ASTM F1843 [7, 8]. If a raw material does not fulfill one of those standards, its physical and chemical properties, including its bulk density, chemical composition, and radioactivity, should be evaluated [7]. In addition, clarification that the material does not undergo chemical change during the manufacturing process should be obtained.

##### Chemical Composition

If a ceramic material contains a metal, either as part of its chemical composition or as an additive material, the purpose and chemical composition of the metal should be clarified.

##### Fine Structure

The fine structure (mean crystal grain size) should be clarified in either a sample of the final product or a test sample prepared under the same conditions as the final product.

##### Surface Treatment

For surface-treated dental implant fixtures or dental implant abutments, the surface treatment method and treatment conditions should be clarified. Surface treatment other than roughening may need to be evaluated separately in consideration of its intended clinical effect. Under visual observation, no abnormality in shape, burr on the surface, scratch, adhesion of foreign matter, coating with foreign matter, or any other visual abnormality should be seen.

##### Stability

The stability of the product should be evaluated [9], and subsequently an appropriate storage method and shelf life should be established. For products that do not require any specific storage method to ensure their quality, or for products that do not deteriorate over time, stability does not need to be evaluated [10].

##### Assurance of Sterility

The sterility of dental implants supplied as sterilized shall be assured on the basis of the appropriate sterilization validation standards or other equivalent or superior standards.

##### Residual Ethylene Oxide Test

The residual gas concentration of dental implants sterilized with ethylene oxide gas should be evaluated [11].

##### Risk Assessment

A risk analysis and assessment, including of any surface treatment residues on dental implant fixtures or dental implant abutments and of the fatigue strength of dental implants, should be performed [12, 13].

#### Safety

##### Biological Safety

The biological safety of the product should be evaluated [6, 14, 15]. The notification categorizes the contact site of dental implants as “implantable” and the contact time as “long-term (permanent),” and evaluation based on these categories is required [6]. In this case, the method of using the product should be fully considered. If the packaging material comes into direct or indirect contact with dental implants via the filling fluid in the package, the evaluation should also examine the influence of such contact.

##### Magnetic Resonance Safety

Magnetic resonance safety should be evaluated as described in the notification of PSEHB/ELD Notification No. 0801-1/PSEHB/SD Notification No. 0801-4 dated August 1, 2019 [16].

#### Physical Performance and Other Requirements

Ceramic materials show elastic, not plastic, deformation, which eliminates the potential for instantaneous brittle fracture at high applied stress levels. However, the physical strength and toughness of the product should still be evaluated. For instance, the bending strength should be 500 MPa or more (2axis bending) or 800 MPa or more (4-point bending) for dental implant abutments and 800 MPa or more (4-point bending) for dental implant fixtures [7].

The fatigue strength of the assembly should be equivalent, or superior, to that of current approved products or otherwise be clinically acceptable. Each final product of a dental implant fixture, dental implant abutment, dedicated abutment screw, and dental implant superstructure material should be analyzed. In addition, a risk assessment should be performed using the whole system constructed from the final products. The final products should have the highest fracture risk in terms of fatigue strength when they are assembled by tightening at the specified torque [17, 18]. For final products assembled from several components individually sterilized or processed/adjusted before use, the final product should be evaluated by using the individual components with the highest fracture risk. In particular, if part of the assembly is an approved product from another manufacturer, the combination shall be subjected to risk assessment by fatigue test. The material should have sufficient impact resistance as a dental implant and should be equivalent, or superior, to the raw material used for the approved product.

Solubility and degradability should be evaluated [19]. The amount dissolved within 16 h should not exceed 50 µg/cm^2^. Osteointegration capacity and the status of adverse events should be confirmed in the canine jaw bone [15].

### Other Matters

For the product, if it is difficult to confirm their equivalence to an approved product for the evaluations explained above, evaluation by supplementary testing, such as by animal testing, should be considered, depending on the characteristics of the product.

#### Matters Related to Nonclinical Studies

Clinical evaluation to demonstrate clinical efficacy and safety is not required if there is no novelty to the approved product in terms of shape, dimension, method of use, etc. However, the safety and efficacy of the product should still be appropriately evaluated, with a focus on equivalence to an approved product (Table 2). If there are medical devices used concomitantly, evaluation in the presence of such devices is necessary.

#### Matters Related to Clinical Studies

If there is novelty in the shape, size, method of use, etc., and evaluation of the efficacy and safety is insufficient or difficult to achieve by nonclinical studies alone, clinical evaluation will be necessary to demonstrate clinical efficacy and safety. If there are medical devices used concomitantly, evaluation in the presence of such devices is also necessary.

Safety should be evaluated by performing medical examinations and tests to confirm that there is no pain, discomfort, change in perception, or sign of infection caused by the dental implant. It should also be evaluated whether the safety of the dental implant is equivalent to that of approved titanium dental implants.

Mobility status, occlusal pain, and amount of bone resorption should be evaluated over time after attachment of the superstructure. With the dental implant fixture in place, it may be acceptable to evaluate the implant stability quotient by resonance frequency analysis and the clinical mobility by using a dynamic periodontal tissue examination/diagnostic device to confirm changes over time in osteointegration. However, evaluation of these parameters alone should not constitute the primary evaluation, and the success or failure of the dental implant treatment should also be evaluated.

## Conclusions

Here, we present a set of principles for premarketing evaluation of the safety and efficacy of zirconia-based ceramic dental implants used to restore functional defects and improve esthetics in individuals with missing teeth. These principles arose from a meeting of experts under the “Project for the Development of a Guideline for the Evaluation of Ceramic Dental Implants” of the Ministry of Health, Labour and Welfare of Japan, and they are the principles that are considered most important at present. There are currently few types of ceramic dental implants approved for use in Japan, and it is hoped that these principles will facilitate the clinical development and approval of emerging ceramic dental implant materials.

We intend to revise this set of principles in the future on the basis of technological innovations and the accumulation of knowledge, and they should not be considered binding in regard to the contents of applications for approval. The evaluations mentioned in this document should be performed on the basis of scientific rationality after fully understanding the characteristics of the product, and with reference to other relevant domestic and international guidelines.

## Data Availability

No datasets were generated or analysed during the current study.
